# Assessment of Trends in the Design, Accrual, and Completion of Trials Registered in ClinicalTrials.gov by Sponsor Type, 2000-2019

**DOI:** 10.1001/jamanetworkopen.2020.14682

**Published:** 2020-08-26

**Authors:** Gillian Gresham, Jill L. Meinert, Arthur G. Gresham, Curtis L. Meinert

**Affiliations:** 1 Department of Medicine, Cedars-Sinai Medical Center, Los Angeles, California; 2 Center for Clinical Trials and Evidence Synthesis, Johns Hopkins University, Baltimore, Maryland

## Abstract

**Importance:**

ClinicalTrials.gov is a valuable resource that can be used to trace the state and nature of trials. Since its launch in 2000, more than 345 000 trials have been registered. Little is known about the characteristics and trends in clinical trials over time and how they differ by sponsor type.

**Objective:**

To assess trends in clinical trials registered in ClinicalTrials.gov over time and by sponsor type.

**Design, Setting, and Participants:**

This cross-sectional study included clinical trials (interventional studies) registered in ClinicalTrials.gov from January 1, 2000, through December 31, 2019. The trials were grouped by lead sponsor: National Institutes of Health (NIH) and other US government agencies, industry, and other sources (foundations, universities, hospitals, clinics, and others). A static version of the Clinical Trials Transformation Initiative Aggregate Analysis of ClinicalTrials.gov database was downloaded on January 1, 2020, for analysis.

**Main Outcomes and Measures:**

ClinicalTrials.gov registration fields, including overall status, phase, intervention, number of sites, use of masking and randomization, sample size, and time to study completion by start year and lead sponsor (organization that provided funding or support for a clinical study).

**Results:**

A total of 245 999 clinical trials (interventional studies) were started between 2000 and 2019, of which 135 144 (54.9%) were completed. Among completed trials, 5113 (3.8%) were sponsored by the NIH or a US government agency, 48 668 (36.0%) by industry, and 81 363 (60.2%) by other sources. Most trials were single center (61.3%), randomized (65.6%), and phase 1 to 2 (35.5%) or did not have a US Food and Drug Administration–defined phase (38.4%), with fewer drug trials being conducted over time. Sample sizes were small (median, 60; interquartile range [IQR], 30-160) and diminished over time. Trial median completion times varied by lead sponsor: 3.4 years (IQR, 1.9-5.0 years) for NIH- and US government–sponsored trials, 1.2 years (IQR, 0.5-2.4 years) for industry trials, and 2.1 years (IQR, 1.1-3.7) for trials sponsored by other sources.

**Conclusions and Relevance:**

The findings suggest that the composition and design of trials changed from 2000 to 2019 and differed substantially by sponsor type. Increased funding toward larger randomized clinical trials may be warranted to inform clinical decision-making and guide future research.

## Key Points

**Question:**

What are the characteristics and trends of clinical trials registered in ClinicalTrials.gov over time, and how do they differ by sponsor type?

**Findings:**

In this cross-sectional study of ClinicalTrials.gov registration data on 245 999 interventional studies started between 2000 and 2019 that were sponsored by the National Institutes of Health or other US government agencies, industry, or other sources (foundations, universities, hospitals, clinics, and others), most trials were small, single-site studies that did not have US Food and Drug Administration–defined phases and were sponsored by other sources. Median sample sizes and years to trial completion decreased over time.

**Meaning:**

The findings suggest that the composition and design of trials changed between 2000 and 2019 and differed substantially by sponsor type; increased funding toward larger randomized clinical trials may be warranted to inform clinical decision-making and guide future research.

## Teaser

This cross-sectional study assesses characteristics and trends in clinical trials registered in ClinicalTrials.gov from 2000 to 2019 and how they differed by sponsor type.

## Introduction

Since ClinicalTrials.gov was launched in 2000, more than 345 000 interventional and observational studies have been registered.^1,2,3^ ClinicalTrials.gov is managed by the National Library of Medicine and is an online resource for health care professionals, researchers, patients, and the general public. It is an important resource that can be used to view and access clinical trials registration data. Analyzing clinical trials metadata can illuminate important trends over time, such as the composition, size, design, and types of trials being funded.

There have been updates to the clinical trials registration and reporting requirements since implementation of the US Food and Drug Administration (FDA) Modernization Act of 1997, which mandated clinical trials registration and led to the establishment of ClinicalTrials.gov.^4,5^ In 2005, the International Committee of Medical Journal Editors (ICMJE) required registration of clinical trials as a prerequisite for publication.^6^ Subsequently, the FDA Act (FDAAA 801) of 2007 expanded requirements to the types of trials being registered, key data elements being entered, and basic results being reported.^7^ The Final Rule became effective in January 2017, further clarifying and expanding on the registration and requirements of FDAAA 801.^8^ Some changes include the types of trials subject to the requirements, the information that must be submitted and data elements that are required to be entered on registration, and additional results information reporting requirements for trials.^8^ Simultaneously, a policy was issued by the National Institutes of Health (NIH) to require registration and results reporting for all trials funded by the NIH regardless of whether the trials are covered by the FDAAA 801 requirements of the Final Rule.^8^

Availability of the Clinical Trials Transformation Initiative Aggregate Analysis of ClinicalTrials.gov (CTTI AACT) database has facilitated and improved the ability to analyze ClinicalTrials.gov registration data.^9^ In 2017, the CTTI AACT database was upgraded to a cloud-based platform that allows for open access to the complete set of trials registered in ClinicalTrials.gov for download and analysis. Its restructured and relational format facilitates analysis and provides access to additional fields that are not readily available in direct exports from ClinicalTrials.gov.

Previous reports of ClinicalTrials.gov registration data have focused analyses on specific funders, such as the NIH; on a single condition; or on a particular registration element within ClinicalTrials.gov.^10,11,12^ To our knowledge, no studies have characterized trials by sponsor type during this 20-year time span. Thus, our objective was to assess the characteristics and trends of clinical trials started from January 1, 2000, through December 31, 2019, and to compare trends by sponsor type.

## Methods

### Study Design and Setting

This cross-sectional study included clinical trials (interventional studies) with start dates between January 1, 2000, and December 31, 2019, that were registered in ClinicalTrials.gov and accessed using the CTTI AACT database.^13^ Observational studies and studies with expanded access were excluded from the analysis. CTTI AACT is a relational cloud-based database that includes aggregated and restructured data from ClinicalTrials.gov. Content is updated daily and can be publicly accessed using pgAdmin (pgAdmin Development Team), R (R Foundation for Statistical Computing), SAS (SAS Institute Inc), or pSQL (PostgreSQL Global Development Group). Characterization of the CTTI AACT content and navigation through the CTTI AACT database followed definitions from the publicly available CTTI AACT comprehensive data dictionary^14^ and definitions available in ClinicalTrials.gov. A static version of the CTTI AACT database was downloaded for analysis on January 1, 2020. This is an analysis of publicly available aggregate trial data; thus, institutional review board approval was not required. This study followed the Strengthening the Reporting of Observational Studies in Epidemiology (STROBE) reporting guideline.

### Variables of Interest

ClinicalTrials.gov registration fields, as coded in the CTTI AACT database, included the following: trial start and completion dates; study type (interventional or observational); overall status (completed, withdrawn, terminated, suspended, open to enrollment, recruiting, not yet recruiting, or status unknown); enrollment number; study phase (early phase 1, phase 1, phase 1 to 2, phase 2, phase 3, phase 4, and trials that do not have an FDA-defined phase [phase not applicable (NA)]); treatment assignment (randomized or not randomized); masking (open label or masked); facilities (single center or multicenter); posted results; and lead sponsor (NIH or other US government agency, industry, and all other sponsors). Lead sponsor is defined in ClinicalTrials.gov as the “organization or person who initiates the study and who has authority and control over the study.”^1^ This variable is not the same as funder type, which is derived from multiple data elements in ClinicalTrials.gov and is not available as a discrete field in the database download. Additional calculated variables included time to completion (calculated as the difference between actual completion date and start date for completed trials) and times to posted results. Anticipated and actual enrollment counts were also assessed by comparing target sample size provided at trial registration with the sample size provided on trial completion. A description of each variable as defined in ClinicalTrials.gov and used for the purpose of this article is available in eTable 1 in the Supplement.

### Statistical Analysis

Results were grouped by lead sponsor and start date in 5-year periods: 2000 to 2004, 2005 to 2009, 2010 to 2014, and 2015 to 2019. These year groupings align with changes in the registration and reporting regulations, including the launch of ClinicalTrials.gov, the ICMJE edict, and implementation of FDAAA 801. Trial start dates were used to classify periods because registration dates can be entered retrospectively and thus are more likely to be inaccurate or lead to time misclassification.

Multivariable regression models were fitted to evaluate the association between sample size and sponsor type and were adjusted for start year and other trial design characteristics. An interaction term between start year and lead sponsor was included in the model in which a significant result would indicate an interactive effect. Anticipated and actual sample sizes were compared across sponsor types for trials started and completed between 2010 and 2019 using the available CTTI AACT archived databases for each year. Median times to completion were calculated from start date to actual completion date for completed trials. A 2-sided *P* < .05 was considered to be statistically significant. All tabulations and analyses were duplicated (A.G.G. and J.L.M.) using postgreSQL and SAS. The postgreSQL codes used to generate tables are available in the eAppendix in the Supplement. Additional analyses were performed using Stata, version 15 (StataCorp LLC).

## Results

There were 325 860 registrations on ClinicalTrials.gov as of January 1, 2020, of which 245 999 were clinical trials (interventional studies) started between 2000 and 2019; 135 144 trials (54.9%) were completed (Figure). Overall, there were 8023 NIH- or US government–sponsored trials (3.3%), 70 329 industry-sponsored trials (28.5%), and 167 647 trials sponsored by other funding sources (68.1%). Among the NIH- and US government–sponsored trials, 63.7% were completed, 11.4% were incomplete, 20.2% were active, and 4.6% had unknown status (Table 1). Industry-sponsored trials had the highest percentage of completed trials (69.2%) and the lowest percentage of active trials (16.8%), whereas trials sponsored by other sources had the lowest completion rates (48.5%) and the highest percentage of active trials (29.8%), including trials that were not yet recruiting, were recruiting, were enrolling by invitation, or were active and not recruiting. The number of NIH- and US government–sponsored trials started each year decreased over time, in contrast to the number of trials started per year that were sponsored by industry and other funding sources, which increased over time (eTable 2 in the Supplement).

**Figure.**
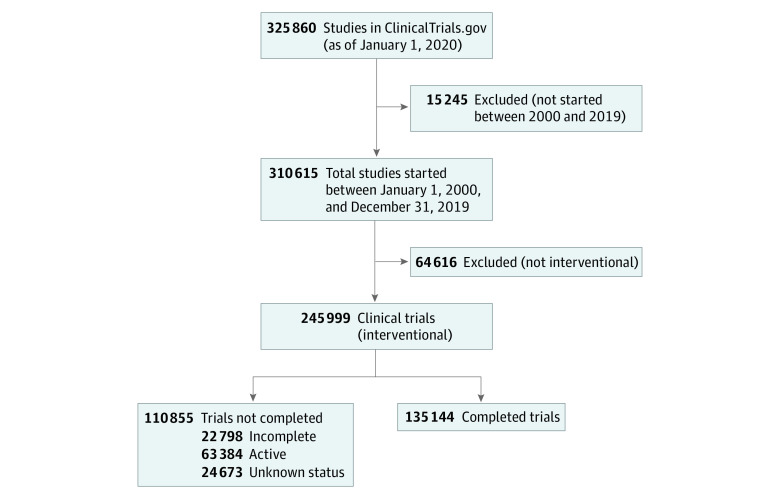
Flow Diagram Incomplete indicates terminated, suspended, or withdrawn, and active indicates recruiting, not yet recruiting, active, or enrolling by invitation.

**Table 1.** Status of the 245 999 Trials Published From 2000 Through 2019 by Lead Sponsor

Start year, lead sponsor	Trials, No. (%)				
	Total	Completed	Incomplete	Active	Unknown
2000-2004					
NIH or US government	2077	1784 (85.9)	172 (8.3)	22 (1.0)	99 (4.8)
Industry	6772	6130 (90.5)	507 (7.5)	6 (0.1)	129 (1.9)
Other	9954	7884 (79.2)	995 (10.0)	214 (2.1)	861 (8.6)
Total	18 803	15 798 (84.0)	1674 (8.9)	242 (1.3)	1089 (5.8)
2005-2009					
NIH or US government	2246	1740 (77.5)	290 (12.9)	87 (3.9)	129 (5.7)
Industry	19 088	16 057 (84.1)	2426 (12.7)	131 (0.7)	474 (2.5)
Other	30 690	21 423 (69.8)	4243 (13.8)	925 (3.0)	4099 (13.4)
Total	52 024	39 220 (75.4)	6959 (13.4)	1143 (2.2)	4702 (9.0)
2010-2014					
NIH or US government	1895	1171 (61.8)	318 (16.8)	311 (16.4)	95 (5.0)
Industry	21 648	16 905 (78.1)	2675 (12.4)	1043 (4.8)	1025 (4.7)
Other	51 761	31 702 (61.2)	5491 (10.6)	5220 (10.1)	9348 (18.1)
Total	75 304	49 778 (66.1)	8484 (11.3)	6574 (8.7)	10 468 (13.9)
2015-2019					
NIH or US government	1805	418 (23.2)	134 (7.4)	1204 (66.7)	49 (2.7)
Industry	22 821	9576 (42.0)	1762 (7.7)	10 615 (46.5)	868 (3.8)
Other	75 242	20 354 (27.1)	3785 (5.0)	43 606 (58.0)	7497 (10.0)
Total	99 868	30 348 (30.4)	5681 (5.7)	55 425 (55.5)	8414 (8.4)
2000-2019					
NIH or US government	8023	5113 (63.7)	914 (11.4)	1624 (20.2)	372 (4.6)
Industry	70 329	48 668 (69.2)	7370 (10.5)	11 795 (16.8)	2496 (3.5)
Other	167 647	81 363 (48.5)	14 514 (8.7)	49 965 (29.8)	21 805 (13.0)
Total	245 999	135 144 (54.9)	22 798 (9.3)	63 384 (25.8)	24 673 (10.0)

Abbreviation: NIH, National Institutes of Health.

Design characteristics of completed trials ordered by lead sponsors and start year are given in Table 2. Most trials were single center (61.3%), randomized (65.6%), open label (55.7%), phase 1 to 2 (35.5%), or lacking an FDA-defined phase (38.4%). Percentages of completed trials that were double-masked, multisite, and randomized were 31.4% for industry-sponsored trials, 12.3% for NIH- and US government–sponsored trials, and 11.0% for other trials and remained stable over time. The overall percentage of drug trials completed decreased from 2000 to 2019 (70.5% in 2000-2004, 61.8% in 2005-2009, 48.9% in 2010-2014, and 40.0% in 2015-2019). This finding is in contrast to a doubling of trials that involved nondrug interventions from 2000 through 2004 (29.6%) to 2015 through 2019 (60.0%). These trends are reflected in the decreasing number of phase 1 to 2 and phase 3 to 4 trials being completed and the increasing number of trials lacking an FDA-defined phase (phase NA). The NIH and US government agencies were the lead sponsors for a larger percentage of completed phase 1 to 2 trials, whereas industry was the lead sponsor for more phase 3 to 4 trials completed over time. Trials sponsored by other sources involved mostly trials lacking an FDA-defined phase. More industry-sponsored trials were multicenter (65.6%) compared with NIH- and US government–sponsored trials (34.2%) and trials sponsored by other sources (27.7%).

**Table 2.** Design Characteristics of the 135 144 Completed Trials by Lead Sponsor and Start Year

Start year, characteristic	Completed trials, No. (%)			
	NIH or US government	Industry	Other	Total
2000-2004				
Facilities				
Multisite	449 (25.2)	4575 (74.6)	2736 (34.7)	7760 (49.1)
Single site	1335 (74.8)	1555 (25.4)	5148 (65.3)	8038 (50.9)
Allocation				
Randomized	841 (47.1)	4346 (70.9)	4949 (62.8)	10 136 (64.2)
Nonrandomized	149 (8.4)	1260 (20.5)	1178 (14.9)	2587 (16.4)
NA	794 (44.5)	524 (8.5)	1757 (22.3)	3075 (19.4)
Masking				
Masked	469 (26.3)	2756 (45.0)	2847 (36.1)	6072 (38.4)
Open label	906 (50.8)	3227 (52.6)	4479 (56.8)	8612 (54.5)
Missing	409 (22.9)	147 (2.4)	558 (7.1)	1114 (7.1)
Interventions				
Drug	1095 (61.4)	5369 (87.6)	4666 (59.2)	11 130 (70.5)
Other	686 (38.6)	761 (12.4)	3218 (40.8)	4668 (29.6)
Phase^a^				
1-2	1156 (64.8)	2549 (41.6)	3127 (39.7)	6832 (43.3)
3-4	281 (15.8)	3323 (54.2)	2495 (31.6)	6099 (38.6)
NA	347 (19.5)	258 (4.2)	2262 (28.7)	2867 (18.2)
Sample size^b^				
<100	1023 (63.3)	2408 (41.4)	4303 (59.2)	7734 (52.6)
100-1000	497 (30.8)	2972 (51.1)	2593 (35.7)	6062 (41.2)
>1000	96 (5.9)	440 (7.6)	368 (5.1)	904 (6.2)
Multisite, randomized, and masked	135 (7.6)	2172 (35.4)	826 (10.5)	3133 (19.8)
Total	1784 (100)	6130 (100)	7884 (100)	15 798 (100)
2005-2009				
Facilities				
Multisite	603 (34.7)	11 025 (68.7)	5667 (26.5)	17 295 (44.1)
Single site	1137 (65.3)	5032 (31.3)	15 756 (73.5)	21 925 (55.9)
Allocation				
Randomized	1013 (58.2)	11 036 (68.7)	14 573 (68.0)	26 622 (67.9)
Nonrandomized	221 (12.7)	2834 (17.7)	2742 (12.8)	5797 (14.8)
NA	506 (29.1)	2187 (13.6)	4108 (19.2)	6801 (17.3)
Masking				
Masked	617 (35.5)	7917 (49.3)	9369 (43.7)	17 903 (45.7)
Open label	1013 (58.2)	7972 (49.7)	11 845 (55.3)	20 830 (53.1)
Missing	110 (6.3)	168 (1.0)	209 (1.0)	487 (1.2)
Interventions				
Drug	993 (57.1)	12 832 (79.9)	10 409 (48.6)	24 234 (61.8)
Other	747 (42.9)	3225 (20.1)	11 014 (51.4)	14 986 (38.2)
Phases^a^				
1-2	1053 (60.5)	8503 (53.0)	6603 (30.8)	16 159 (41.2)
3-4	219 (12.6)	6382 (39.7)	5906 (27.6)	12 507 (31.9)
NA	468 (26.9)	1172 (7.3)	8914 (41.6)	10 554 (26.9)
Sample size^b^				
<100	1150 (66.3)	8243 (51.7)	13 931 (65.6)	23 324 (60.0)
100-1000	509 (29.3)	6908 (43.4)	6483 (30.5)	13 900 (35.7)
>1000	76 (4.4)	781 (4.9)	812 (3.8)	1669 (4.3)
Multisite, randomized, and masked	238 (13.7)	5487 (34.2)	2123 (9.9)	7848 (20.0)
Total	1740 (100)	16 057 (100)	21 423 (100)	39 220 (100)
2010-2014				
Facilities				
Multisite	412 (35.2)	10 423 (61.7)	8665 (27.3)	19 500 (39.2)
Single site	759 (64.8)	6482 (38.3)	23 037 (72.7)	30 278 (60.8)
Allocation				
Randomized	747 (63.8)	11 228 (66.4)	22 576 (71.2)	34 551 (69.4)
Nonrandomized	154 (13.1)	1877 (11.1)	2585 (8.2)	4616 (9.3)
NA	270 (23.1)	3800 (22.5)	6541 (20.6)	10 611 (21.3)
Masking				
Masked	487 (41.6)	8098 (47.9)	15 319 (48.3)	23 904 (48.0)
Open label	665 (56.8)	8759 (51.8)	16 257 (51.3)	25 681 (51.6)
Not reported	19 (1.6)	48 (0.3)	126 (0.4)	193 (0.4)
Interventions				
Drug	509 (43.5)	12 422 (73.5)	11 410 (36.0)	24 341 (48.9)
Other	662 (56.5)	4483 (26.5)	20 292 (64.0)	25 437 (51.1)
Phases^a^				
1-2	632 (54.0)	9488 (56.1)	6829 (21.5)	16 949 (34.1)
3-4	95 (8.1)	5122 (30.3)	6581 (20.8)	11 798 (23.7)
NA	444 (37.9)	2295 (13.6)	18 292 (57.7)	21 031 (42.2)
Sample size^b^				
<100	817 (69.8)	9991 (59.2)	21 173 (66.9)	31 981 (64.3)
100-1000	317 (27.1)	6317 (37.4)	9321 (29.5)	15 955 (32.1)
>1000	36 (3.1)	574 (3.4)	5104 (25.1)	1771 (3.6)
Multisite, randomized, and masked	177 (15.1)	5066 (30.0)	3795 (12.0)	9038 (18.2)
Total	1171 (100)	16 905 (100)	31 702 (100)	49 778 (100)
2015-2019				
Facilities				
Multisite	285 (31.8)	4752 (49.6)	4729 (23.2)	9686 (31.9)
Single site	133 (68.2)	4824 (50.4)	15 625 (76.8)	20 662 (68.1)
Allocation				
Randomized	308 (73.7)	6311 (65.9)	14 797 (72.7)	21 416 (70.6)
Nonrandomized	37 (8.8)	842 (8.8)	1625 (8.0)	2504 (8.3)
NA	73 (17.5)	2423 (25.3)	3932 (19.3)	6428 (21.2)
Masking				
Masked	222 (53.1)	4740 (49.5)	10 260 (50.4)	15 222 (50.2)
Open label	196 (46.9)	4822 (50.4)	10 038 (49.3)	15 056 (49.6)
Not reported	0 (0.0)	14 (0.2)	56 (0.3)	70 (0.2)
Interventions				
Drug	127 (30.4)	6680 (69.8)	5322 (26.2)	12 129 (40.0)
Other	291 (69.6)	2896 (30.2)	15 032 (73.8)	18 219 (60.0)
Phase^a^				
1-2	186 (44.5)	5316 (55.5)	2532 (12.4)	8034 (26.5)
3-4	28 (6.7)	2092 (21.9)	2708 (13.3)	4828 (15.9)
NA	204 (48.8)	2168 (22.6)	15 114 (73.3)	17 486 (57.6)
Sample size^b^				
<100	311 (74.4)	6419 (67.1)	14 653 (72.0)	21 383 (70.5)
100-1000	97 (23.2)	2916 (30.5)	5104 (25.1)	8117 (26.8)
>1000	10 (2.4)	230 (2.4)	590 (2.9)	830 (2.7)
Multisite, randomized, and masked	77 (18.4)	2549 (26.6)	2212 (10.9)	4838 (15.9)
Total	418 (100)	9576 (100)	20 354 (100)	30 348 (100)
2000-2019				
Facilities				
Multisite	1597 (34.2)	30 847 (65.6)	21 797 (27.7)	54 241 (38.7)
Single site	3516 (67.8)	17 821 (34.4)	59 566 (72.3)	80 903 (61.3)
Allocation				
Randomized	2909 (56.8)	32 921 (63.9)	56 895 (66.7)	92 725 (65.6)
Nonrandomized	561 (12.0)	6813 (14.2)	8130 (10.1)	15 504 (11.4)
NA	1643 (31.3)	8934 (21.9)	16 338 (23.2)	26 915 (23.1)
Masking				
Masked	1795 (35.0)	23 511 (45.7)	37 795 (42.7)	63 101 (43.3)
Open label	2780 (57.7)	24 780 (53.6)	42 619 (56.4)	70 179 (55.7)
Not reported	538 (7.3)	377 (0.7)	949 (0.88)	1864 (1.0)
Interventions				
Drug	2724 (34.4)	37 303 (76.7)	31 807 (13.8)	71 834 (53.2)
Other	2389 (65.6)	11 365 (23.3)	49 556 (86.2)	63 308 (46.9)
Phase^a^				
1-2	3027 (59.2)	25 856 (53.1)	19 091 (23.5)	47 974 (35.5)
3-4	623 (12.2)	16 919 (34.8)	17 690 (21.7)	35 232 (26.1)
NA	1463 (28.6)	5893 (12.1)	44 582 (54.8)	51 938 (38.4)
Sample size^b^				
<100	3301 (66.8)	27 061 (56.1)	54 060 (67.2)	84 422 (63.2)
100-1000	1420 (28.8)	19 113 (39.7)	23 501 (29.2)	44 034 (32.9)
>1000	218 (4.4)	2025 (4.2)	2931 (3.6)	5174 (3.9)
Multisite, randomized, and masked	627 (12.3)	15 274 (31.4)	8956 (11.0)	24 857 (18.4)
Total	5113 (100)	48 668 (100)	81 363 (100)	135 144 (100)

Abbreviations: NA, not applicable; NIH, National Institutes of Health.

^a^ Phase 1 to 2 includes early phase 1, phase 1, phase 1 to 2, and phase 2 trials. Phase 3 to 4 includes phase 2 to 3, phase 3, and phase 4 trials. Phase NA is defined as trials without a US Food and Drug Administration–defined phase, including trials of devices or behavioral interventions.

^b^ Actual and anticipated sample sizes. Tabulations exclude missing enrollment data.

Median sample sizes for completed trials by sponsor and phase over time are given in Table 3. The overall median sample size for trials between 2000 and 2019 was 60 individuals (interquartile range [IQR], 30-160 individuals) and decreased between 2000 and 2019 for all sponsors. Sample sizes were largest for industry-sponsored trials, with a median of 75 individuals (IQR, 32-236 individuals) compared with NIH- and US government–sponsored trials (median, 55 individuals; IQR, 28-140 individuals) and trials funded by other sources (median, 58 individuals; IQR, 28-128 individuals) (Table 3). Trial sample sizes were less than 100 individuals in 56.1% of industry-sponsored trials compared with 66.8% of NIH- and US government–sponsored trials and 67.2% of trials sponsored by other sources overall.

**Table 3.** Sample Sizes for the 135 144 Completed Trials by Lead Sponsor and Phase Over Time

Start year, lead sponsor	Sample size, median (IQR)			
	Phase 1-2^a^	Phase 3-4^b^	Phase NA^c^	All trials
2000-2004				
NIH/US government	48 (29-85)	223 (80-727)	150 (50-400)	60 (32-157)
Industry	51 (30-120)	300 (122-600)	109 (36-341)	140 (48-400)
Other	42 (24-78)	120 (50-338)	99 (40-243)	68 (32-195)
Total	47 (27-92)	210 (80-500)	100 (40-277)	86 (38-266)
2005-2009				
NIH/US government	44 (24-83)	150 (49-400)	98 (30-257)	55 (26-139)
Industry	48 (26-108)	258 (106-538)	72 (35-173)	91 (36-273)
Other	38 (20-67)	85 (40-206)	70 (32-172)	60 (29-140)
Total	42 (24-87)	150 (60-400)	70 (32-178)	66 (30-190)
2010-2014				
NIH/US government	36 (20-69)	149 (64-480)	70 (33-206)	48 (25-124)
Industry	42 (24-90)	251 (101-513)	60 (30-140)	66 (30-204)
Other	33 (18-64)	74 (40-162)	62 (30-153)	59 (28-130)
Total	39 (20-77)	120 (50-325)	62 (30-152)	60 (29-150)
2015-2019				
NIH/US government	37 (20-72)	111 (61-357)	53 (28-123)	47 (25-100)
Industry	39 (22-74)	247 (105-515)	46 (23-100)	53 (25-140)
Other	30 (15-60)	70 (36-134)	52 (27-109)	50 (25-104)
Total	36 (19-70)	110 (50-291)	51 (26-108)	51 (25-114)
2000-2019^d^				
NIH/US government	42 (24-80)	180 (64-506)	89 (34-253)	55 (28-140)
Industry	45 (24-95)	261 (108-540)	57 (28-131)	75 (32-236)
Other	36 (20-66)	80 (40-198)	60 (30-145)	58 (28-128)
Total	40 (22-80)	140 (56-379)	60 (30-146)	60 (30-160)

Abbreviations: IQR, interquartile range; NA, not applicable; NIH, National Institutes of Health.

^a^ Includes early phase 1, phase 1, phase 1 to 2, and phase 2 trials.

^b^ Includes phase 2 to 3, phase 3, phase 3 to 4, and phase 4 trials.

^c^ Phase NA is defined as trials without a US Food and Drug Administration–defined phase, including trials of devices or behavioral interventions.

^d^ Totals exclude missing data. Enrollment data are missing for 1514 records.

In multivariable regression of completed trials (eTable 3 in the Supplement), sample sizes decreased by 8.2 persons every 5 years. Phase 1 to 2 trials decreased by 2.2 persons (95% CI, 1.5-2.9 persons), phase 3 to 4 trials by 8.8 persons (95% CI, 5.3-12.3 persons), and trials lacking an FDA-defined phase by 4.2 persons (95% CI, 0.9-7.5 persons) every 5 years. Comparing sample sizes by sponsor, NIH- and US government–sponsored trials were smaller than industry-sponsored trials for phase 1 to 2 trials (−2.5; 95% CI, −4.0 to 1.0; *P* < .001), phase 3 to 4 trials (−82.7; 95% CI, −96.4 to −69.0; *P* < .001), and overall (−12.7; 95% CI, −14.9 to −10.6; *P* < .001). Interaction terms for start year and sponsor were statistically significant (eTable 3 in the Supplement). Planned sample sizes at the beginning of the trial were larger than actual sample sizes when trials were completed across all phases and sponsor types (eTable 4 and eFigure 3 in the Supplement).

Median times to trial completion by lead sponsor were 3.4 years (IQR, 1.9-5.0 years) for NIH- and US government–sponsored trials, 1.2 years (IQR, 0.5-2.4 years) for industry trials, and 2.1 years (IQR, 1.1-3.7 years) for trials sponsored by other sources between 2000 and 2019 (eFigure 1 in the Supplement). Table 4 shows median times to completion and IQRs for completed trials by sponsor type and start year, which decreased over time (eFigure 2 in the Supplement). For example, median years to completion for phase 3 to 4 trials sponsored by the NIH and other US government agencies were 5.4 (IQR, 3.7-7.7) in 2000, 3.8 (IQR, 2.3-5.9) in 2005, 3.7 (IQR, 2.9-4.9) in 2010, and 3.2 (IQR, 1.9-3.7) in 2015. Times to completion for industry-sponsored phase 3 to 4 trials remained relatively steady over time: 3.2 years (IQR, 1.9-5.2 years) in 2000, 1.7 years (IQR, 0.9-2.7 years) in 2005, 1.7 years (IQR, 0.9-3.1 years) in 2010, and 1.6 years (IQR, 0.9-2.5 years) Times to completion for phase 3 to 4 trials sponsored by other sources were 6.0 years (IQR, 3.9-9.1 years) in 2000, 3.1 years (IQR, 1.8-5.0 years) in 2005, 3.0 years (IQR, 1.6-4.4 years) in 2010, and 1.6 years (IQR, 0.9-2.5 years) in 2015.

**Table 4.** Time to Trial Completion for the 135 144 Completed Trials by Lead Sponsor Over Time^a^

Start year	Time to trial completion, median (IQR), y								
	Phase 1-2			Phase 3-4			Phase NA^b^		
	NIH or US government	Industry	Other	NIH or US government	Industry	Other	NIH or US government	Industry	Other
2000	3.8 (1.9-5.7)	2.3 (1.0-5.1)	5.4 (3.5-7.6)	5.4 (3.7-7.7)	3.2 (1.9-5.2)	6.0 (3.9-9.1)	4.6 (3.0-5.9)	3.7 (1.5-9.3)	5.3 (3.6-7.7)
2001	3.7 (2.2-5.9)	1.8 (0.7-3.5)	5.7 (3.4-8.2)	4.9 (3.2-5.9)	2.5 (1.6-4.2)	5.1 (3.3-7.5)	4.2 (2.9-5.4)	4.9 (2.3-8.5)	4.7 (2.9-7.1)
2002	4.0 (2.7-6.1)	1.2 (0.2-2.7)	5.2 (3.0-7.5)	4.6 (3.0-6.0)	2.0 (1.1-3.3)	4.4 (2.8-6.3)	3.8 (2.7-5.3)	1.0 (0.2-4.0)	4.0 (2.7-5.8)
2003	3.7 (2.4-5.9)	1.5 (0.4-3.0)	4.7 (2.9-6.9)	4.3 (2.4-5.7)	1.8 (1.1-3.1)	4.0 (2.4-5.7)	3.1 (2.2-4.9)	2.5 (0.2-5.4)	3.9 (2.4-5.8)
2004	4.0 (2.5-6.4)	1.4 (0.4-2.7)	4.4 (2.7-6.2)	3.0 (2.0-5.2)	1.7 (1.0-2.8)	3.3 (2.1-5.1)	3.2 (2.1-4.5)	1.0 (0.1-3.7)	3.4 (1.9-5.2)
2005	3.8 (2.0-5.5)	1.4 (0.5-2.8)	3.9 (2.2-6.0)	3.8 (1.8-5.8)	1.7 (0.9-2.7)	3.1 (1.8-5.0)	3.0 (2.0-4.3)	1.7 (0.3-5.2)	2.9 (1.6-4.9)
2006	3.9 (2.1-5.9)	1.4 (0.6-2.8)	3.8 (2.3-5.9)	3.2 (1.5-5.5)	1.7 (1.0-2.7)	2.9 (1.8-4.5)	3.1 (1.8-4.7)	2.2 (0.6-4.8)	2.7 (1.4-4.5)
2007	4.0 (2.3-6.1)	1.3 (0.5-2.7)	3.6 (1.9-5.6)	3.7 (1.6-5.6)	1.6 (0.9-2.7)	2.7 (1.6-4.4)	3.4 (1.9-4.8)	1.8 (0.4-3.8)	2.7 (1.3-4.2)
2008	4.0 (2.4-5.8)	1.0 (0.4-2.3)	3.4 (1.9-5.2)	3.5 (1.9-5.2)	1.6 (0.8-2.7)	2.6 (1.4-4.3)	3.1 (1.5-4.4)	1.2 (0.3-3.0)	2.6 (1.4-4.5)
2009	3.3 (1.3-5.6)	1.0 (0.3-2.4)	3.3 (1.8-5.2)	3.9 (2.3-5.3)	1.7 (0.9-3.1)	2.5 (1.3-4.0)	3.5 (2.2-5.3)	1.2 (0.5-2.8)	2.6 (1.3-4.2)
2010	3.6 (1.9-5.0)	0.9 (0.3-2.2)	3.1 (1.7-4.8)	3.7 (2.9-4.9)	1.7 (0.9-2.9)	2.4 (1.2-3.9)	3.0 (1.6-4.4)	1.3 (0.5-3.5)	2.4 (1.2-3.9)
2011	3.5 (2.0-5.0)	1.0 (0.3-2.2)	2.7 (1.5-4.3)	3.5 (2.3-4.4)	1.7 (1.0-2.9)	2.3 (1.3-3.7)	3.2 (1.8-4.4)	1.3 (0.6-3.0)	2.2 (1.1-3.7)
2012	3.3 (1.7-4.6)	1.1 (0.3-2.2)	2.6 (1.4-4.1)	3.2 (1.8-4.7)	1.7 (1.0-2.8)	2.2 (1.2-3.5)	3.2 (1.9-4.6)	1.2 (0.6-2.7)	2.2 (1.1-3.7)
2013	3.5 (2.0-4.9)	1.0 (0.3-2.2)	2.5 (1.3-3.8)	3.7 (2.7-4.3)	1.7 (0.9-2.8)	2.1 (1.2-3.2)	3.1 (1.9-4.2)	1.3 (0.5-2.6)	2.0 (1.1-3.3)
2014	2.7 (1.7-3.9)	0.9 (0.3-2.0)	2.0 (1.1-3.2)	2.5 (1.5-4.0)	1.6 (0.9-2.6)	1.8 (1.0-2.9)	3.3 (1.6-4.2)	1.3 (0.5-2.4)	1.8 (1.0-2.4)
2015	2.6 (1.6-3.6)	0.9 (0.3-1.9)	1.7 (0.9-2.8)	3.2 (1.9-3.7)	1.6 (0.9-2.4)	1.6 (0.9-2.5)	2.2 (1.1-3.3)	1.3 (0.5-2.6)	1.6 (0.9-2.5)
2016	1.8 (1.1-2.4)	0.8 (0.4-1.6)	1.6 (0.9-2.3)	1.6 (0.8-2.8)	1.4 (0.9-2.2)	1.4 (0.8-2.1)	2.0 (1.0-2.4)	1.3 (0.5-2.4)	1.3 (0.7-2.0)
2017	1.7 (1.1-2.1)	0.7 (0.3-1.2)	1.3 (0.8-1.8)	1.2 (1.1-1.2)	1.2 (0.7-1.7)	1.1 (0.7-1.6)	1.5 (0.7-1.9)	1.0 (0.5-1.9)	1.0 (0.6-1.5)
2018	0.8 (0.6-1.3)	0.5 (0.2-0.8)	0.8 (0.4-1.1)	1.4 (1.4-1.4)	0.7 (0.5-1.0)	0.7 (0.4-1.0)	0.9 (0.6-1.1)	0.7 (0.3-1.5)	0.6 (0.4-1.0)
2019	0.6 (0.6-0.6)	0.2 (0.1-0.4)	0.3 (0.1-0.5)	NR	0.2 (0.1-0.5)	0.4 (0.2-0.5)	0.2 (0.1-0.5)	0.6 (0.3-1.2)	0.3 (0.2-0.5)
All	3.5 (1.9-5.3)	1.0 (0.3-2.2)	2.9 (1.5-4.8)	3.8 (2.3-5.4)	1.7 (0.9-2.7)	2.3 (1.3-3.9)	3.0 (1.7-4.4)	1.0 (0.3-2.2)	1.7 (0.9-3.1)

Abbreviations: IQR, interquartile range; NIH, National Institutes of Health; NR, not reported.

^a^ Trends in time to trial completion should be interpreted with caution within the past 3 to 5 years because fewer trials were completed and trials may not have had sufficient time to be completed. Data are presented for descriptive purposes.

^b^ Phase NA is defined as trials without a US Food and Drug Administration–defined phase, including trials of devices or behavioral interventions.

From 2007, when posting results became required, to 2019, the percentages of completed trials posting results by agency were 47.7% for NIH- and US government-sponsored trials, 37.8% for industry-sponsored trials, and 16.0% for trials sponsored by other sources. The median times to posting were 1.4 years (IQR, 0.9-3.0 years) for all trials, 1.3 years (IQR, 0.9-2.9 years) for industry-sponsored trials, 1.6 years (IQR, 1.0-3.3 years) for NIH- and US government-sponsored trials, and 1.6 years (IQR, 0.9-3.1 years) for trials sponsored by other sources.

## Discussion

ClinicalTrials.gov is an important resource that can be used to characterize the state and nature of trials. We describe trends and characteristics of 245 999 trials that were registered in ClinicalTrials.gov and started between 2000 and 2019. We found that trials had smaller sample sizes and were being completed in less time and that most trials were sponsored by other sources (foundations, universities, hospitals, clinics, and others) from 2000 to 2019.

### Observed Trends in Clinical Trial Characteristics Over Time

The number of trials started per year increased between 2000 and 2019, with the largest increase observed in the number of trials started each year by other sources. A similar trend was observed for industry-sponsored trials started per year, whereas the number of NIH- and US government-sponsored trials started per year decreased. Part of the decrease may have been associated with differential uptake of registration across sponsor types, which was faster for NIH- and US government-sponsored trials, within the first 5 years of its launch, accounting for most new registrations.

Differences were observed in clinical trial design characteristics over time, including different distributions across trial phases, intervention types, use of randomization or masking, and number of centers involved over time. There was a decrease in the percentages of phase 3 to 4 trials and drug trials being conducted over time compared with an increase in the percentages of nondrug trials and trials without an FDA-defined phase. The rate in difference between early-phase trials (phase 1-2) and phase 3 trials decreased by almost half by the end of 2019. This shift may be explained by the increased uptake in registration for these trials, expansion of the clinical trial definition, or increasing interest in other intervention types (eg, behavioral interventions, imaging, biologic, and devices) in recent years.^15,16,17^ The decreasing percentages of trials that involved drugs may also be associated with increasing costs and complexity of conducting phase 3 to 4 drug trials.

Overall, trial sample sizes decreased over time and took less time to complete. Median times to trial completion varied by sponsor type and phase. Industry completed trials at faster rates compared with the NIH and US government and other funders, possibly in association with more efficient trial startup processes and higher recruitment rates. Reasons for this trend may include changes in the types of outcomes being used (eg, surrogate outcomes and biomarkers as well as patient-reported outcomes), increasing trial-associated costs, and greater budget constraints. With an overall median sample size of 60 persons per trial, the ability to generate meaningful, reproducible differences with such a sample size remains questionable.^10,18^ Reports from almost 10 years ago had similar conclusions, without any evidence of change or improvement.^10,12,19^ The original planned sample sizes were not met and were often smaller compared with the actual sample size when the trial was completed.^20^ Reasons for not achieving the planned sample size, other than meeting the scientific goals of the trial, include recruitment and retention difficulties, business decisions, and unavailability or discontinuation of funding.^21,22,23^ At time of analysis, there were 21 455 trials started in 2019 and registered in ClinicalTrials.gov. If we assume registrations in ClinicalTrials.gov account for 70% of all trials registered and that the median cost (direct and indirect) per trial from start to finish is $1 000 000 at a minimum, the total cost for trials in 2019 would be approximately $31 billion. The median cost for comparative efficacy trials (phase 3-4) is closer to $19 million per trial, with larger trials ranging up to $53 million per trial.^24,25^ Thus, increased funding for larger randomized clinical trials may be warranted to inform clinical decision-making and answer important clinical and health policy questions.

### Opportunities for Improvement

The findings suggest that registration and reporting systems could be further improved. There appears to be a need for ClinicalTrials.gov to modify its registration system to accommodate the broader range of trials being conducted and the collaborative arrangements involved. For instance, there is currently no explicit data element for funding source in ClinicalTrials.gov. Thus, the lead sponsor variable was used to estimate trends by the different agencies and organizations as classified in ClinicalTrials.gov. The term *lead sponsor* refers to the primary organization that oversees study implementation and is responsible for conducting data analysis and is further used to determine the primary funding source for the study.^1^ The impetus for ClincalTrials.gov was the FDA. That history is reflected in the emphasis on drug trials, US funding, and intermixing sponsor as funder and holder of Investigational New Drug applications. Because trials are largely collaborative and can involve several funders, there is potential for misclassification, underreporting, or overreporting of estimates of funding sources. Although methods have been described to estimate the probable funding source from ClinicalTrials.gov, this information cannot be easily exported or analyzed using the publicly available data set or derived without extensive data manipulation and assumptions.^10^ Thus, an explicit data element for the primary source or sources of funding with potential linkage to the study project number (eg, NIH Report Expenditures and Results Tool [RePORTER] for NIH-funded trials) and the total amount and duration of funding, if available, may improve the ability to compare trials across funding sources, reducing the risk of misclassification of trials in the other funder categories.

Additional updates that may be beneficial to the analysis of ClinicalTrials.gov registration data include further subdivisions for trial phases beyond the FDA-defined phases to reduce the number of trials lacking an FDA-defined phase, expansion of the trial typography as listed in ClinicalTrials.gov to better capture the different types of trial designs, discrete elements for the outcome types (eg, time to event, surrogate, composite, and patient reported) and for specific time points for the primary outcomes for analysis purposes, and reduction of free-text renders to prevent formatting issues when analyzing the database. This approach would allow for future comparisons of specific outcome types and adjust analyses by outcome duration. Improvements to the system for tracking publications related to the trial are needed, including a separate field for the publication associated with the primary outcome, to determine the fraction of trials registered that are published. Publication is the sine qua non of trials, but only a fraction of completed trials are published. Thus, continued efforts to enforce the timely and complete reporting of results are important to reduce the reporting biases associated with delayed publication or failure to publish.^26,27,28,29,30^

### Limitations

This study has limitations. The analysis was limited to the available data as registered in ClinicalTrials.gov and thus may not provide a complete or accurate assessment of the clinical trials research enterprise. The ClinicalTrials.gov database structure and the individual study records have evolved since ClinicalTrials.gov was first launched in 2000, affected by the different registration and reporting requirements over time. Changes in required registration fields, data formats, and key definitions and terms (eg, clarification of applicable clinical trial and changes in phase categories) made it difficult to compare characteristics registered over time and resulted in missing data encountered for certain registration fields, such as enrollment, number of facilities, or overall status.^12,32^ ClinicalTrials.gov is only 1 of multiple registration sites where trials can be registered. The World Health Organization International Clinical Trials Registry Platform has 16 other places where trials can be registered with varying analytic capabilities. Although trials are required to meet ICMJE standards, it is difficult to obtain a single comprehensive evaluation of all registered trials. It also remains unclear how many duplicate registrations may exist, especially for non-US studies that may be registered in both ClinicalTrials.gov and a second or third registration registry.^31^ Thus, whether trials registered in ClinicalTrials.gov are representative of trials registered elsewhere may remain unknown until there is a system for merging registries into 1 or for establishing a universal and standardized data system for harvesting trial information from all registries through the World Health Organization International Clinical Trials Registry Platform.

## Conclusions

Even with its limitations, ClinicalTrials.gov registration provides valuable insights into the massive clinical trials research enterprise. The findings suggest that the composition and design of trials changed over time and differed substantially by sponsor type. Increased funding toward larger randomized clinical trials may be warranted to inform clinical decision-making and guide future research.

## Notes

Supplement. **eTable 1.** Selected Terms and Definitions Used on ClinicalTrials.gov **eTable 2.** Number of Trials Started by Year Between 2000 and 2019 **eTable 3.** Predictors of Sample Size for Completed Trials in Multivariable Regression by Phase **eTable 4.** Anticipated and Actual Sample Sizes for Trials Started and Completed Between 2010 Through 2019 by Lead Sponsor and Phase **eFigure 1.** Time to Study Completion by Lead Sponsor **eFigure 2.** Median Time (Years) to Trial Completion by Lead Sponsor and Phase **eFigure 3.** Anticipated vs Actual Sample Size of Trials Started and Completed Between 2010 Through 2019 by Lead Sponsor and Year **eAppendix.** Example postgreSQL Code to Generate CT.gov Registration Dataset Used for Analysis
