# Supplementary material for: Assessment of Trends in the Design, Accrual, and Completion of Trials Registered in ClinicalTrials.gov by Sponsor Type, 2000-2019
Source: JAMA Netw Open. 2020 Aug 26;3(8):e2014682. doi: 10.1001/jamanetworkopen.2020.14682 (PMC7450351; doi:10.1001/jamanetworkopen.2020.14682)
Supplement: Supplement. — eTable 1. Selected Terms and Definitions Used on ClinicalTrials.gov eTable 2. Number of Trials Started by Year Between 2000 and 2019 eTable 3. Predictors of Sample Size for Completed Trials in Multivariable Regression by Phase eTable 4. Anticipated and Actual Sample Sizes for Trials Started and Completed Between 2010 Through 2019 by Lead Sponsor and Phase eFigure 1. Time to Study Completion by Lead Sponsor eFigure 2. Median Time (Years) to Trial Completion by Lead Sponsor and Phase eFigure 3. Anticipated vs Actual Sample Size of Trials Started and Completed Between 2010 Through 2019 by Lead Sponsor and Year eAppendix. Example postgreSQL Code to Generate CT.gov Registration Dataset Used for Analysis [file jamanetwopen-3-e2014682-s001.pdf]

Supplementary Online Content

Gresham G, Meinert JL, Gresham AG, Meinert CL. Assessment of trends in the design, accrual, and completion of trials registered in ClinicalTrials.gov by sponsor type, 2000-2019. *JAMA Netw Open.* 2020;3(8):e2014682. doi:10.1001/jamanetworkopen.2020.14682

eTable 1. Selected Terms and Definitions Used on ClinicalTrials.gov

eTable 2. Number of Trials Started by Year Between 2000 and 2019

eTable 3. Predictors of Sample Size for Completed Trials in Multivariable Regression by Phase

eTable 4. Anticipated and Actual Sample Sizes for Trials Started and Completed Between 2010 Through 2019 by Lead Sponsor and Phase

eFigure 1. Time to Study Completion by Lead Sponsor

eFigure 2. Median Time (Years) to Trial Completion by Lead Sponsor and Phase

eFigure 3. Anticipated vs. Actual Sample Size of Trials Started and Completed Between 2010 Through 2019 by Lead Sponsor and Year

eAppendix. Example postgreSQL Code to Generate CT.gov Registration Dataset Used for Analysis

This supplementary material has been provided by the authors to give readers additional information about their work.

© 2020 Gresham G et al. *JAMA Network Open.*

eTable 1. Selected terms and definitions used on ClinicalTrials.gov

Term	*ClinicalTrials.gov Definition*
**Clinical Study**	*A research study involving human volunteers (also called participants) that is intended to add to medical knowledge. There are two types of clinical studies: interventional studies (also called clinical trials) and observational studies.*
**Interventional Study (clinical trial)**	*A type of clinical study in which participants are assigned to groups that receive one or more intervention/treatment (or no intervention) so that researchers can evaluate the effects of the interventions on biomedical or health-related outcomes. The assignments are determined by the study's protocol. Participants may receive diagnostic, therapeutic, or other types of interventions.*
**Observational study**	*A type of clinical study in which participants are identified as belonging to study groups and are assessed for biomedical or health outcomes. Participants may receive diagnostic, therapeutic, or other types of interventions, but the investigator does not assign participants to a specific interventions/treatment.*
**ClinicalTrials.gov identifier (NCT number)**	*The unique identification code given to each clinical study upon registration at ClinicalTrials.gov. The format is "NCT" followed by an 8-digit number (for example, NCT00000419).*
**Funder type**	*Describes the organization that provides funding or support for a clinical study. This support may include activities related to funding, design, implementation, data analysis, or reporting. Organizations listed as sponsors and collaborators for a study are considered the funders of the study.*
**Sponsor**	*The organization or person who initiates the study and who has authority and control over the study.*
**Collaborator**	*An organization other than the sponsor that provides support for a clinical study. This support may include activities related to funding, design, implementation, data analysis, or reporting.*
**Phase**	*The stage of a clinical trial studying a drug or biological product, based on definitions developed by the U.S. Food and Drug Administration (FDA). The phase is based on the study's objective, the number of participants, and other characteristics. There are five phases: Early Phase 1 (formerly listed as Phase 0), Phase 1, Phase 2, Phase 3, and Phase 4. Not Applicable is used to describe trials without FDA-defined phases, including trials of devices or behavioral interventions.*
**Phase 1**	*A phase of research to describe clinical trials that focus on the safety of a drug. They are usually conducted with healthy volunteers, and the goal is to determine the drug's most frequent and serious adverse events and, often, how the drug is broken down and excreted by the body. These trials usually involve a small number of participants.*
**Phase 2**	*A phase of research to describe clinical trials that gather preliminary data on whether a drug works in people who have a certain condition/disease (that is, the drug's effectiveness). For example, participants receiving the drug may be compared to similar participants receiving a different treatment, usually an inactive substance (called a placebo) or a different drug. Safety continues to be evaluated, and short-term adverse events are studied.*
**Phase 3**	*A phase of research to describe clinical trials that gather more information about a drug's safety and effectiveness by studying different populations and*

	different dosages and by using the drug in combination with other drugs. These studies typically involve more participants.
**Phase 4**	*A phase of research to describe clinical trials occurring after FDA has approved a drug for marketing. They include postmarket requirement and commitment studies that are required of or agreed to by the study sponsor. These trials gather additional information about a drug's safety, efficacy, or optimal use.*
**Phase Not Applicable**	*Describes trials without FDA-defined phases, including trials of devices or behavioral interventions.*

*****All definitions transcribed from the ClinicalTrials.gov glossary available at: :

https://clinicaltrials.gov/ct2/about-studies/glossary

© 2020 Gresham G et al. *JAMA Network Open.*

eTable 2. Number of trials started by year between 2000 and 2019

Year started	NIH/US Gov	Industry	Other	Total
2000	320	454	1,087	1,861
2001	358	742	1,290	2,390
2002	426	1,358	1,699	3,483
2003	421	1,806	2,531	4,758
2004	552	2,412	3,347	6,311
2005	501	2,855	4,363	7,719
2006	527	3,574	5,186	9,287
2007	413	3,940	6,034	10,387
2008	403	4,344	7,091	11,838
2009	402	4,375	8,016	12,793
2010	457	4,261	8,621	13,339
2011	407	4,397	9,303	14,107
2012	341	4,254	10,291	14,886
2013	355	4,203	11,152	15,710
2014	335	4,533	12,394	17,262
2015	373	4,572	13,566	18,511
2016	350	4,528	14,690	19,568
2017	342	4,370	15,136	19,848
2018	357	4,656	15,473	20,486
2019	383	4,695	16,377	21,455
**Total**	**8,023**	**70,329**	**167,647**	**245,999**

© 2020 Gresham G et al. *JAMA Network Open.*

eTable 3. Predictors of sample size for completed trials in multivariable regression by phase

	All Trials (N=103,300)		Phase 1-2 (N=39,119)		Phase 3-4 (N=30,017)		Phase N/A (N=34.163)	
Variable	Regression coefficient (95% CI)	P-value	Regression coefficient (95% CI)	P-value	Regression coefficient (95% CI)	P-value	Regression coefficient (95% CI)	P-value
Year started (every 5 years)	-8.2 (-9.1, -7.3)	<0.001	-2.2 (-2.9, -1.5)	<0.001	-8.8 (-12.3,- 5.3)	<0.001	-4.2 (-7.5, -0.90)	0.01
Lead sponsor:								
Industry	REF		REF		REF		REF	
NIH/U.S. Gov	-12.7 (-14.9, -10.6)	<0.001	-2.5 (-4.0, -1.0)	<0.001	-82.7 (-96.4, -69.0)	<0.001	-2.2 (-6.6, 2.2)	0.33
Other	-11.2 (-12.2, -10.2)	<0.001	-6.2 (-7.0, -5.4)	<0.001	-114.2 (-118.7, -109.7)	<0.001	-7.0 (-9.4, -4.7)	<0.001
Year*sponsor:								
Industry	REF		REF		REF		REF	
NIH/U.S. Gov	-1.5 (-4.1, 1.0)	0.24	-3.1 (-5.8, -2.3)	<0.001	-14.0 (-32.6-2.7)	<0.097	-3.4 (-9.1, 2.3)	0.24
Other	3.8 (2.6, 5.0)	<0.001	-0.5 (-1.5, 0.5)	0.3	-7.7 (-12.6, -2.7)	<0.001	-2.1 (-5.6, 1.4)	0.23
Multisite	25.1 (24.2, 26.0)	<0.001	14.3 (13.5, 15.0)	<0.001	63.3 (59.1, 67.6)	<0.001	17.0 (15.4, 18.6)	<0.001
Randomized	42.3 (41.4, 4.8)	<0.001	--[3]	--	94.7 (59.1, 67.6)	<0.001	30.6 (29.0, 32.2)	<0.001

[1]Robust standard error regression to account for sample size with 95% Confidence Intervals (CI)
[2]For trials that started before 2015 to allow for sufficient time to completion
[3]Randomized design not included in model, as this is not a common feature in early-phase (phase 1/2) trial designs.

© 2020 Gresham G et al. *JAMA Network Open.*

eTable 4. Anticipated and actual sample sizes for trials started and completed between 2010 through 2019 by lead sponsor and phase

Phase	Funder	N	Anticipated Sample Size when started Median (IQR)		Actual Sample Size when completed Median (IQR)	
All trials	US gov't	1,555	66	(36-160)	49	(25-121)
	Industry	29,950	80	(36-232)	61	(28-184)
	Other	51,465	60	(30-150)	55	(26-120
	Total	79,000	68	(32-180)	57	(27-135)
Phase 1-2	US gov't	752	50	(30-95)	37	(20-72)
	Industry	9, 834	50	(28-100)	41	(24-84)
	Other	7,376	40	(20-70)	31	(17-62)
	Total	17,962	45	(24-90)	38	(20-75)
Phase 3-4	US gov't	122	190	(80-456)	139	(63-451)
	Industry	5,906	280	(120-534)	252	(103-519)
	Other	6,653	90	(44-200)	71	(39-151)
	Total	12,667	150	(60-370)	119	(50-315)
Phase N/A	US gov't	553	100	(50-240)	62	(30-192)
	Industry	2,529	60	(30-150)	52	(25-120)
	Other	21,162	72	(38-180)	60	(30-130)
	Total	24,244	72	(36-180)	59	(29-129)

© 2020 Gresham G et al. *JAMA Network Open.*

eFigure 1. Time to study completion by lead sponsor

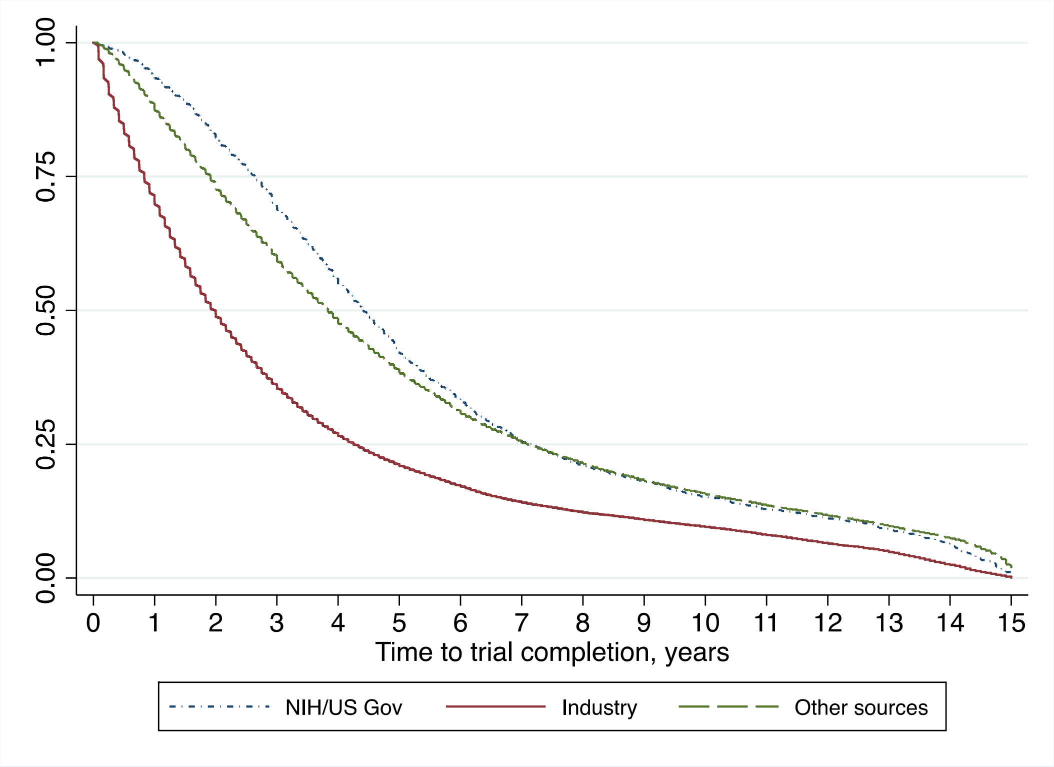

Kaplan-Meier Survival Curve of percent completed (y axis) by time to trial completion (x axis), calculated from start date to primary completion date among completed trials.

© 2020 Gresham G et al. *JAMA Network Open.*

eFigure 2. Median time (years) to trial completion for completed trials by lead sponsor and phase

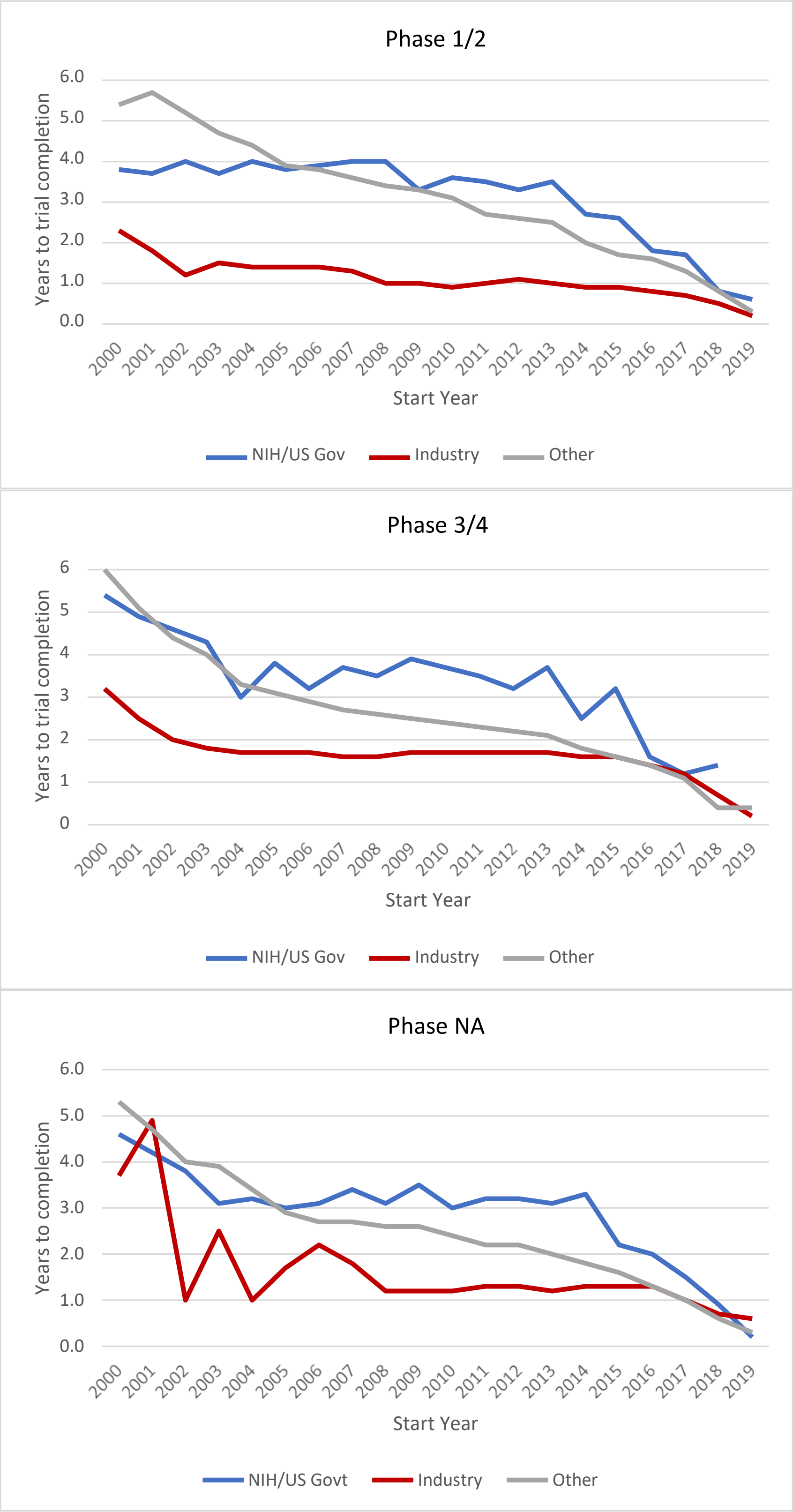

© 2020 Gresham G et al. *JAMA Network Open.*

eFigure3. Anticipated versus actual sample size of trials started and completed between 2010 through 2019, by lead sponsor and year

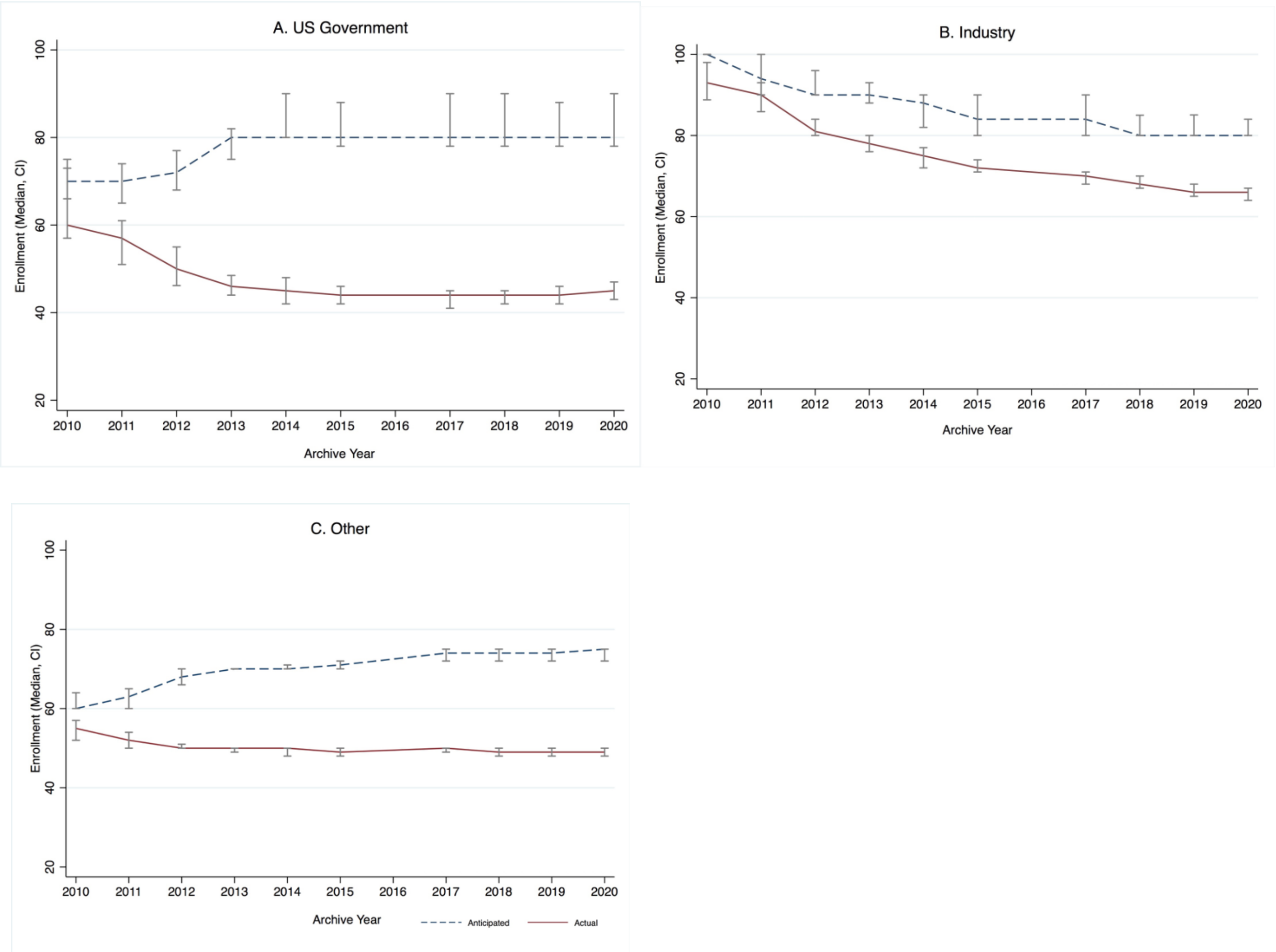

*Archive year: A version of the AACT CTTI static database from each year available (2010-2020) as downloaded and sample sizes were compared between when trial was first registered (anticipated) and when trial was completed (actual). In 2016, AACT CTTI database was converted from Oracle to PostgreSQL, thus gap in enrollment data.

© 2020 Gresham G et al. *JAMA Network Open.*

eAppendix. Example postgreSQL code to generate CT.gov registration dataset used for analysis

a- Sample query using AACT database

```
Server [localhost]: aact-db.ctti-clinicaltrials.org
Database [postgres]: aact
Port [5433]: 5432
Username [postgres]: greshamg
Password for user greshamg:
psql (10.5, server 11.1)
WARNING: psql major version 10, server major version 11.
         Some psql features might not work.
Type "help" for help.

aact=> select count(distinct a.nct_id), a.agency_class, b.phase
aact->                  from sponsors a join studies b
aact->                     on b.nct_id = a.nct_id
aact->                             join calculated_values c
aact->                     on c.nct_id=a.nct_id
aact->
aact->                where b.overall_status = 'Completed'
aact->                and b.study_type = 'Interventional'
aact->                and extract(year from b.primary_completion_date) between 2006 and 2018
aact->                group by agency_class,
aact->                b.phase
aact->                order by agency_class,
aact->                b.phase
aact->                ;
```

b- Code to create AACT registration dataset

```
 create table t_cohort1 as
(  select
   a.nct_id
 , a.overall_status
 , case when a.overall_status = 'Completed'                              then 'Completed'
        when a.overall_status in ('Terminated', 'Withdrawn', 'Suspended') then 'Stopped'
        when a.overall_status in (   'Recruiting'
                                   , 'Not yet recruiting'
                                   , 'Active, not recruiting'
                                   , 'Enrolling by invitation')           then 'Active'
        else  a.overall_status   -- overall_status = 'Unknown Status' or null or some new status
   end as overall_status_grp
 , a.phase
 , case when a.phase in ('N/A') then 'N/A'
        else                        'Phase' end as phase_grp_cat
 , case when a.phase in ('Phase 1','Early Phase 1', 'Phase 1/Phase 2', 'Phase 2')   then 'Phase_1_2'
        when a.phase in ('Phase 2/Phase 3', 'Phase 3','Phase 4')                    then 'Phase_3_4'
        else a.phase
   end as phase_grp
 , a.study_type
 , b.lead_or_collaborator
 , b.agency_class
 , case when b.agency_class in ('NIH', 'U.S. Fed') then 'US_Fed_or_NIH'
        else b.agency_class
   end as agency_class_grp
 , start_date
 , extract(year from a.start_date)      as year_Started
--  , case when extract(year from a.start_date) in (2004,2005,2006,2007,2008) then 'Yr_2004_2008'
--         when extract(year from a.start_date) in (2009,2010,2011,2012,2013) then 'Yr_2009_2013'
--         when extract(year from a.start_date) in (2014,2015,2016,2017,2018) then 'Yr_2014_2018'
--         else extract(year from a.start_date)::text
--    end as year_started_grp
```

© 2020 Gresham G et al. *JAMA Network Open.*

```
  , start_date_type
  , completion_date
  , case when completion_date - start_date                                          <= 0365 then '01
year  or    less'
         when completion_date - start_date > 0365 and completion_date - start_date  <= 0730 then '01
year  to 02 years'
         when completion_date - start_date > 0730 and completion_date - start_date  <= 1095 then '02
years to 03 years'
         when completion_date - start_date > 1095 and completion_date - start_date  <= 1460 then '03
years to 04 years'
         when completion_date - start_date > 1460 and completion_date - start_date  <= 1826 then '04
years to 05 years'
         when completion_date - start_date > 1826 and completion_date - start_date  <= 2191 then '05
years to 06 years'
         when completion_date - start_date > 2191 and completion_date - start_date  <= 2556 then '06
years to 07 years'
         when completion_date - start_date > 2556 and completion_date - start_date  <= 2921 then '07
years to 08 years'
         when completion_date - start_date > 2921 and completion_date - start_date  <= 3287 then '08
years to 09 years'
         when completion_date - start_date > 3287 and completion_date - start_date  <= 3652 then '09
years to 10 years'
         when completion_date - start_date > 3652 and completion_date - start_date  <= 4017 then '10
years to 11 years'
         when completion_date - start_date > 4017 and completion_date - start_date  <= 4382 then '11
years to 12 years'
         when completion_date - start_date > 4382 and completion_date - start_date  <= 4748 then '12
years to 13 years'
         when completion_date - start_date > 4748 and completion_date - start_date  <= 5113 then '13
years to 14 years'
         when completion_date - start_date > 5113 and completion_date - start_date  <= 5478 then '14
years to 15 years'
         when completion_date - start_date > 5478 and completion_date - start_date  <= 5843 then '15
years to 16 years'
         else (completion_date - start_date)::text
    end  as time_to_completion_grp
  , extract(year from a.completion_date) as year_Completed
--  , case when extract(year from a.completion_date) in (2004,2005,2006,2007,2008) then 'Yr_2004_2008'
--         when extract(year from a.completion_date) in (2009,2010,2011,2012,2013) then 'Yr_2009_2013'
--         when extract(year from a.completion_date) in (2014,2015,2016,2017,2018) then 'Yr_2014_2018'
--         else extract(year from a.completion_date)::text
--     end as year_completed_grp
  , completion_date_type
  , primary_completion_date
  , primary_completion_date_type
  , last_update_submitted_date
  , results_first_posted_date
  , results_first_posted_date_type

  , enrollment
  , case when enrollment > 0     then 'enrollment data exists'
         when enrollment = 0     then 'enrollment data zero'
         when enrollment is null then 'enrollment data null'
         else enrollment::text
     end as enrollment_grp
  , case when enrollment >   0  and enrollment <=   50  then 'enrollment 001 to 0050'
         when enrollment >  50  and enrollment <=  100  then 'enrollment 051 to 0100'
         when enrollment > 100  and enrollment <=  500  then 'enrollment 101 to 0500'
         when enrollment > 500  and enrollment <= 1000  then 'enrollment 501 to 1000'
         when enrollment > 1000                         then 'enrollment   over 1000'
     end as enrollment_count_grp
  , why_stopped
  , c.allocation
  , case
         when c.allocation  is null                            then '(null)'
         when c.allocation  in ('Randomized', 'Random Sample') then 'Random'
         when c.allocation  =  'Non-Randomized'                then 'Non-Random'
         else c.allocation
     end as allocation_grp
  , c.masking
  , case when c.masking in (
```

© 2020 Gresham G et al. *JAMA Network Open.*

```
             'Triple'
          ,  'Quadruple'
          ,  'Single'
          ,  'Double'
          ) then 'Masked 1/2/3/4'
          else c.masking
          end as masking_grp
  , d.were_results_reported
  , d.months_to_report_results
  , d.has_single_facility
  , f.number_of_facilities
  , case when f.number_of_facilities > 0     then 'facilities data exists'
         when f.number_of_facilities is null then 'facilities data null'
         else f.number_of_facilities::text
     end as facilities_grp
  , case when f.number_of_facilities >   0 and  f.number_of_facilities <=   1 then 'facilities
001'
         when f.number_of_facilities >   1 and  f.number_of_facilities <=  10 then 'facilities  002 to
010'
         when f.number_of_facilities >  10 and  f.number_of_facilities <= 100 then 'facilities  011 to
100'
         when f.number_of_facilities > 100                                    then 'facilities    over
100'
      end as facilities_count_grp
  , g.gender
  , case when g.gender = 'Female'         then 'Female Only'
         when g.gender in ('Male', 'All') then 'Male / All'
         when ( g.gender is null or g.gender = '')  then 'gender data null or blank'
         else g.gender
     end as gender_grp
  , i.number_of_interventions      as number_of_interventions
  , i.number_of_intervention_types as number_of_intervention_types
  , i.intervention_type_list::text as intervention_type_list
  , case
         when     lower(i.intervention_type_list::text) like '%null%'
              and i.number_of_intervention_types = 1    then '(null)'
         when     lower(i.intervention_type_list::text) like '%drug%'
              and i.number_of_intervention_types = 1    then 'Drug'
         when     lower(i.intervention_type_list::text) like '%drug%'
              and i.number_of_intervention_types > 1    then 'Drug and Other'
         when     lower(i.intervention_type_list::text) not like '%drug%'
                                                        then 'Other'
         else  i.intervention_type_list::text
         end  as intervention_types_grp
  , bcc.number_of_browse_conditions      as number_of_browse_conditions
  , bcc.mesh_term_list                   as mesh_term_list
  , case
         when     lower(bcc.mesh_term_list::text)    is null then '(null)'
         when     lower(bcc.mesh_term_list::text)    like '%carcinoma%'
           or     lower(bcc.mesh_term_list::text)    like '%leukemia%'
           or     lower(bcc.mesh_term_list::text)    like '%neoplasm%'
           or     lower(bcc.mesh_term_list::text)    like '%cancer%'
           or     lower(bcc.mesh_term_list::text)    like '%lymphoma%'     then 'Cancer'
    --     when     lower(bcc.mesh_term_list::text)    like '%coronary%'
    --       or     lower(bcc.mesh_term_list::text)    like '%lung%'         then 'Heart/Lung'
    --    when     lower(bcc.mesh_term_list::text)    like '%hiv%'          then 'HIV'
    --    when     lower(bcc.mesh_term_list::text)    like '%kidney%'       then 'Kidney'
    --    when     lower(bcc.mesh_term_list::text)    like '%stroke%'       then 'Stroke'
    --    when     lower(bcc.mesh_term_list::text)    like '%depressi%'     then 'Depression'
    --    when     lower(bcc.mesh_term_list::text)    like '%diabetes%'     then 'Diabetes'
    --    when     lower(bcc.mesh_term_list::text)    like '%arthrit%'      then 'Arthritis'
    --    when     lower(bcc.mesh_term_list::text)    like '%infection%'    then 'Infection'
              else      'Other'
     end                                 as mesh_term_list_grp
  , cc.number_of_conditions
  , cc.name_list as conditions_name_list
  , case
          when     lower(cc.name_list::text)    is null    then '(null)'
          when     lower(cc.name_list::text)    like '%healthy%'    then 'Healthy'
          when     lower(cc.name_list::text)    like '%carcinoma%'
            or     lower(cc.name_list::text)    like '%leukemia%'
```
© 2020 Gresham G et al. *JAMA Network Open.*

```
            or     lower(cc.name_list::text)    like '%neoplasm%'
            or     lower(cc.name_list::text)    like '%cancer%'
            or     lower(cc.name_list::text)    like '%lymphoma%'   then 'Cancer'
           else    'Other'
     end                                 as conditions_name_list_grp

  from studies a
           join sponsors b on
           a.nct_id = b.nct_id
           join designs c on
           a.nct_id = c.nct_id
           join calculated_values d on
           a.nct_id = d.nct_id
 left outer
           join facilities_counts f on
           a.nct_id = f.nct_id
           join eligibilities g on
           a.nct_id = g.nct_id
 left outer
           join intervention_type_lists i on
           a.nct_id = i.nct_id
 left outer
           join browse_conditions_counts bcc on
           a.nct_id = bcc.nct_id
 left outer
           join conditions_name_lists cc on
           a.nct_id = cc.nct_id
  where b.lead_or_collaborator = 'lead'
  -- and overall_status = 'Completed'
   and study_type = 'Interventional'
 --  and (    extract(year from start_date)      between 2004 and 2018
 --      )
)
;
alter table t_cohort1
  add primary key (nct_id);
vacuum full t_cohort1 ;
```

© 2020 Gresham G et al. *JAMA Network Open.*
